# Incidence of maternal near miss in the public health sector of Harare, Zimbabwe: a prospective descriptive study

**DOI:** 10.1186/s12884-018-2092-7

**Published:** 2018-11-26

**Authors:** Henry Chikadaya, Mugove Gerald Madziyire, Stephen P. Munjanja

**Affiliations:** Department of Obstetrics and Gynaecology, University of Zimbabwe College of Health Sciences, Central Hospital, P O Box ST 14, Southerton, Harare, Zimbabwe

**Keywords:** Maternal near miss, Maternal mortality, Severe maternal morbidity, Severe maternal outcome, Mortality indexs

## Abstract

**Background:**

Maternal ‘near miss’ can be a proxy for maternal death and it describes women who nearly died due to obstetric complications. It measures life threatening pregnancy related complications and allows the assessment of the quality of obstetric care.

**Methods:**

A prospective descriptive study was carried out from October 1 2016 to 31 December 2016, using the WHO criteria for maternal ‘near miss’ at the two tertiary public hospitals which receive referrals of all obstetric complications in Harare city, Zimbabwe. The objective was to calculate the ratio of maternal ‘near miss’ and associated factors. All pregnant women who developed life threatening complications classified as maternal near miss using the WHO criteria were recruited and followed up for six weeks from discharge, delivery or termination of pregnancy or up to the time of death.

**Results:**

During this period there were 11,871 births. One hundred and twenty three (123) women developed severe maternal outcomes, 110 were maternal ‘near miss’ morbidity and 13 were maternal deaths. The maternal ‘near miss’ ratio was 9.3 per 1000 deliveries, the mortality index (MI) was 10.6% and the maternal mortality ratio was 110 per 100,000 deliveries. The major organ dysfunction among cases with severe maternal outcomes (SMO) was cardiovascular dysfunction (76.9%). The major causes of maternal near miss were obstetric haemorrhage (31.8%), hypertensive disorders (28.2%) and complications of miscarriages (20%). The intensive care unit (ICU) admission rate was 7.3 per 100 cases of SMO and 88.8% of maternal deaths occurred without ICU admission.

**Conclusion:**

The MNM ratio was comparable to that in the region. Obstetric haemorrhage was a leading cause of severe maternal morbidity though with less mortality when compared to hypertensive disorders and abortion complications. Zimbabwe should adopt maternal near miss ratio as an indicator for evaluating its maternal health services.

## Background

Maternal morbidity is part of a continuum from maternal good health to maternal mortality. In any setting, women who suffer severe maternal complications share many circumstantial and pathological factors related to their condition [1]. Some women die as a result of these complications, but a proportion of them narrowly escape death either by chance or due to the quality of care they receive [2]. Maternal deaths (MD) can be likened to the ‘tip of an iceberg’ with the base being maternal morbidity, in that more women survive pregnancy complications than the ones who die [3, 4]. The primary indicator of maternal health care and hence quality of obstetric care is Maternal Mortality Ratio (MMR) [5]. By monitoring only maternal deaths at institutions, very few lessons can be learnt to prevent further deaths. This is further compounded by the fact that in maternal death review meetings most of the patient’s records are either missing or incompletely filled [6, 7]. Quality of obstetric care can be assessed using either outcome indicators like maternal mortality ratio, or MNM indicators or using process indicators such as the use of prophylactic antibiotics for the reduction of puerperal sepsis after caesarian section [1, 8].

In 2009, the WHO introduced the concept of ‘maternal near miss’(MNM) for evaluating the quality of care for severe pregnancy complications [1]. A MNM is defined as a case of a woman who nearly died but survived a complication that has occurred during pregnancy, childbirth or within 42 days of termination of pregnancy [1]. Studying MNM is increasingly being recognized as a useful means to improve the quality of obstetric care, particularly in low and middle income countries [2]. It is estimated that for every woman who dies 20 or more survive severe maternal complications as a result of the pregnancy or delivery [9].

The underlying assumptions of WHO MNM concept are that all maternal deaths involve at least one life threatening condition or organ dysfunction and a substantial proportion of women with one or more of these life threatening conditions are those who have severe pregnancy-related complications or receive critical interventions like blood transfusion and ICU admission [1].

The WHO criteria for MNM are based on clinical, laboratory as well as management based proxies for organ or system dysfunction [1]. The WHO recommends that the maternal near miss approach should be considered in national plans for improving maternal health care [10]. Using near miss indicators has several advantages in that it offers a large number of cases that allows a more rapid and precise assessment of quality of care, it has greater acceptability by involved health workers and institutions since death has not occurred, and it offers an opportunity for interviewing the women themselves [11]. Maternal mortality is among the worst performing health indicators in developing countries [7]. The WHO near miss clinical based criteria when used in Tanzania taking into account available resources, had an improved sensitivity (99.5%) and specificity (100%) for detecting maternal deaths [12].

In Zimbabwe maternal mortality remains high, even in urban settings although women in rural areas are at a significantly higher risk of dying from pregnancy complications [13, 14]. The latest maternal mortality ratio in Zimbabwe is 651 per 100,000 live births [15]. The quality of obstetric care is a large driver of poor maternal outcomes. Recently, there has been some studies that sought to address this issue [9, 14, 16–18]. The maternal mortality ratio patterns established using these retrospective studies are often imprecise and make it difficult to monitor trends or progress regarding the targets of improving maternal care [5]. The burden of severe maternal morbidity is unknown in Zimbabwe including in the public health sector of Harare. We therefore sought to assess the burden of severe maternal morbidity by calculating the MNM ratio and describe factors associated with MNM in the public health sector of Harare city.

## Methods

### Aim

We aimed to establish the MNM ratio and factors associated with severe maternal morbidity in the public sector of Harare.

### Research design

A prospective descriptive study was carried out. This was a quantitative design that concentrated in the collection of numerical data on the occurrences of severe maternal outcomes and associated factors.

### Setting

The population of the city of Harare is 1.542 million. The public sector health facilities of Harare are composed of two hospitals, Harare Central Hospital and Parirenyatwa Hospital and 12 local authority clinics which offer maternity services. The two hospitals are the tertiary hospitals which also serve as the teaching units for the University of Zimbabwe. The 12 local authority clinics are located in residential areas. They provide antenatal care, vaginal deliveries and postnatal care to low risk women. If a high risk pregnancy is identified, or there is a need for a caesarean section, or an obstetric emergency arises, referral is made to either of the two hospitals for further care. Women registered for follow up in a particular unit are referred to as having booked their pregnancies. The two tertiary hospitals also receive referrals from rural provinces across Zimbabwe. Private care maternity services are provided by five smaller facilities mainly located near the central business district.

### Participants

The participants were women who delivered or miscarried, or who had antenatal complications leading to a near miss condition according to WHO near miss criteria in the public health sector of Harare City during the period 1st October to 31st December 2016. We excluded women who had been referred from facilities outside Harare.

A target sample size of 96 was determined using a single proportion formula [19], and the incidence of MNM in a study done at Haydom Lutheran Hospital in Tanzania (23.6 per 1000 live births) which had the highest incidence in the region [20].

### Data collection

Identification of cases was carried out through review of all the women admitted into the obstetrics and gynaecology units at the two tertiary hospitals. Data was collected from patient registers and case notes by the principal researcher and two research assistants. Once the patient was treated and in a stable condition, informed consent was sought. The managing clinicians were questioned in case of doubt or missing information. At the local clinics data was obtained on the total number of deliveries. Data was collected from the 1st of October to the 31st of December 2016.

The primary outcome measure was the MNM ratio in the public sector of Harare during the study period. Secondary outcome indicators such as the MMR, Severe Maternal Outcomes (SMO) ratio, and the Mortality Index (MI) were calculated. SMO – refers to a life threatening condition, i.e. organ dysfunction or death hence includes MNM and maternal deaths. SMO ratio (SMOR) is the number of MNM plus MD per 1000 LB. MNM ratio refers to the number of maternal near-miss cases per 1000 live births (MNMR = MNM/LB). These two indicators, SMO ratio and MNM ratio, give an estimate of the amount of care and resources that would be needed in an area or facility. Maternal near-miss mortality ratio (MNM: 1 MD) refers to the ratio between maternal near- miss cases and maternal deaths. Higher ratios indicate better care. MI refers to the number of maternal deaths divided by the sum of women with life-threatening conditions and maternal deaths expressed as a percentage [MI = MD/ (MNM + MD)]. The higher the index the more women with life-threatening conditions die (low quality of care), whereas the lower the index the fewer women with life-threatening conditions die (better quality of care).

Data was entered and cleaned using IBM SPSS statistics data editor version 20. Frequency tables and cross tabulations were produced for the demographic and clinical variables as well as for the underlying causes.

Ethical clearance was sought and obtained from: Harare Central Hospital Ethics Committee (HCHEC 240616/40); The Director, City of Harare Department of Health; The Joint Research Ethics Committee (JREC Ref: 222/16); Medical Research Council of Zimbabwe (MRCZ/B/1130).

## Results

The total number of deliveries in the public sector of Harare was 11,871 during the study period. Most of the deliveries occurred at the local clinics [6475(54%)], while 27% (3185) and 19% (2211) were delivered at Harare and Parirenyatwa hospitals respectively. Of these, 123 women developed SMO of which 13 were maternal deaths and 110 were maternal near misses. The median age for severe maternal outcomes was 28 years. Median parity was two. Most of the women were married, educated up to secondary level and 60.8% were homemakers. Most (80%) were Christians (Table 1). The maternal near miss ratio was 9.3 per 1000 deliveries, 95% confidence interval (C I) of 7.6–11.0 per 1000 deliveries, with a mortality index of 10.6%. For every maternal death, there were 8.5 maternal near miss events. The maternal mortality ratio was 110/100000 live births, 95% C I of 50–170 deaths per 100,000 live births (Table 2). Of the women who developed severe maternal outcomes 66.7% (82) were referred from local clinics, while 24% (30), 8% (10) were booked at Harare and Parirenyatwa hospitals respectively. One woman came from home.

**Table 1:** Demographic data of women with severe maternal outcomes (*n* = 123) at Harare and Parirenyatwa hospitals in October–December 2016

Characteristic	Parameter	Number	%
Age	Median (interquartile range)	28 (10)	
Parity	Median (interquartile range)	2 (2)	
Marital status	Married	117	90.1
	Single (never married)	9	6.9
	Divorced/separated	2	1.5
	Widowed	2	1.5
Level of education	Primary Secondary Tertiary	12 114 4	9.2 87.7 3.1
Occupation	Homemaker Unskilled Peasant farmer Semi- skilled Skilled	79 25 1 22 3	60.8 19.2 0.8 16.9 2.3
Religion	Christianity Traditional Apostolic	104 2 23	80.0 1.5 17.7

**Table 2:** Summary of deliveries and severe maternal outcomes and near miss indicators in women delivering at public health sector institutions of Harare in October–December 2016

Outcomes	Near-miss indicators
Total deliveries	11,871
Severe maternal outcomes (SMO) cases (number)	123
Maternal deaths (n)	13
Maternal near-miss cases (n)	110
Severe maternal outcome ratio (per 1000 deliveries)	10.4
Maternal near-miss ratio (per 1000 deliveries)	9.3CI 95%: (7.6–11.0)
Maternal mortality ratio (per 100,000 live births)	110 CI 95%: (50–170)
Maternal near-miss: mortality ratio	8.5:1
Mortality index	10.6%

The major underlying causes of maternal near miss were obstetric hemorrhage (31.8%), hypertensive disorders (28.2%) and complications of miscarriages (20%) (Table 3). Among maternal deaths hypertensive disorders of pregnancy were the leading cause accounting for four (30.8%) of deaths, followed by medical disorders [3(23.1%)] and obstetric haemorrhage [3(23.1%)]. The major organ dysfunction among cases with severe maternal outcomes was cardiovascular dysfunction (76.9%), Respiratory dysfunction (69.2), haematologic dysfunction (23.1%) and renal dysfunction (15.4%). There were 9 out of 123 cases of severe maternal outcomes admitted into ICU, giving an ICU admission rate of 7.3 cases per 100 cases of SMO and 88.8% of maternal deaths occurred without ICU admission.

**Table 3:** Underlying causes and organ dysfunction in SMO

Underlying causes	MNM	MD	MI
	(*n* = 110)	(*n* = 13)	
	N (%)	N (%)	(%)
Obstetric hemorrhage	35 (31.8)	2 (15.4)	5.4
Hypertensive disorders	31 (28.2)	4 (30.6)	11.4
Abortion related complications	22 (20)	3 (23.1)	12
Pregnancy-related infection	15 (13.6)	1 (7.7)	6.3
Ruptured ectopic pregnancy	11 (10)	0 (0)	0
Medical disorder	4 (3.6)	3 (23.1)	75
Parasuicide/suicide	1 (0.9)	1 (7.7)	50
Unanticipated complications of management	1 (0.9)	0 (0)	0
Organ Dysfunction			
Cardiovascular dysfunction	72 (65.5)	10 (76.9)	
Respiratory dysfunction	23 (20.9)	9 (69.2)	
Coagulation/hematologic dysfunction	12 (10.9)	3 (23.1)	
Renal dysfunction	4 (3.6)	2 (15.4)	
Hepatic dysfunction	3 (2.7)	0 (0)	
Uterine dysfunction/hysterectomy	2 (1.8)	0 (0)	

*SMO* severe maternal outcomes which refers to women with life-threatening conditions and maternal deaths, *MNM* maternal near miss cases, *MD* maternal death cases, *MI* mortality index calculated as number of maternal deaths divided by the sum of women with life-threatening conditions and maternal deaths expressed as a percentage [MI = MD/ (MNM + MD)]

## Discussion

The MNM ratio was 9.3 per 1000 deliveries and the leading cause was obstetric hemorrhage. The MNM ratio was within the range of MNM ratios described by WHO of around 7.5 cases per 1000 deliveries [1]. In a systematic review by Tuncalp et al., the MNM ratio varies depending on the criteria used and is higher in low and medium income countries [21]. Our MNM ratio is higher when compared to the MNM ratio from a South African study, which reported 4.3 per 1000 [22], and lower to that from a study in Tanzania, (23.6 per 1000 live births) [20].. This could be explained firstly by the fact that in this study we used a broader definition of MNM which included complications of miscarriages and ectopic pregnancies unlike the criteria in the South African study, secondly because the Tanzanian study was hospital based.

The MMR in this study was 110 per 100,000 live births which is lower than the national level of 651 per 100,000 live births [15] and a notable decrease from 361 per 100,000 live births in 2010 in the same population [23]. This shows a continued downward trend likely due to increased resource allocation. Most (67%) patients who developed SMO had booked at local clinics i.e. were low risk women. This was similar to findings from the South African study that reported that a significant proportion of serious complications occur in women who are otherwise regarded as low risk [22] highlighting increased vigilance during antenatal clinic visits for apparently low risk women. The leading underlying causes of SMO in this study are similar to the top 5 causes of maternal mortality in Zimbabwe quoted in various studies [9, 17, 23]. This confirms that MNM can be a proxy for MM [1]. The overall MI was 10.6%. which is comparable to the one from a South African study (14%) [22]. The MI was lowest for obstetric haemorrhage (5.4) and high for hypertensive disorders (11.4), abortion complications (12.0) and medical disorders. This is likely because of improved management of obstetric haemorrhage as a result of availability of free blood and in service training in obstetric emergencies funded by aid organizations. However, hemorrhage is still a leading cause of MNM and there is need for continued health system strengthening so as not to lose gains made by wide spread in service training in the management of obstetric emergencies. The MI for medical disorders is distorted by a low number of cases as there were only 4 cases and 3 of them died. A multi-disciplinary approach is however needed when managing patients with medical conditions in pregnancy so as to recognize complications early. While in service training in obstetric emergencies cover all areas there is need to improve the management protocols for hypertensive disorders and post abortion complications. Most deaths as a result of miscarriages were occurring within hours of arrival which suggests the unsafe nature of abortions in the communities. Earlier studies have also reported a higher case fatality rate from hypertensive disorders and this was reported to be due to inadequate case management [23, 24].

The ICU admission rate among SMO is low and most deaths occurred without being admitted into ICU. There is greater demand for ICU at our tertiary hospitals from non-obstetric cases as well as referrals from rural provinces. There is need to have an ICU dedicated to the maternity units alone. There is also need to better equip hospital wards to have high dependency units with adequate human and material resources so as to improve patient outcome.

### Strengths and weakness

#### Strengths

This study was prospective in nature with every case of severe maternal outcome providing a full complete account of events as outlined in the questionnaire and hence avoiding problems associated with missing records in retrospective studies. We used a validated standard data collection tool and criteria for near miss morbidity that had been used elsewhere in similar low resource settings [11, 12, 20, 22, 25]. Our study was population based which avoids erroneous figures associated with institutional based data due to concentration of complicated cases.

#### Weaknesses

Data collection was based at the two tertiary hospitals and we did not capture cases that crossed over to or were completely managed in the private sector. This number will be low however as there are only 5 private hospitals in Harare with a capacity to attend to obstetric complications and their combined delivery capacity is about 6544/year versus about 50,000 per year for the tertiary hospitals [23]. This was a cross sectional study done over a 3 month period and hence can miss morbidity or mortality patterns due to seasonal trends.

## Conclusion

The MNM ratio of 9.3 per thousand deliveries was comparable to other reports in the region. The mortality index was 10.6%, which is high. Obstetric haemorrhage was a leading cause of severe maternal morbidity though with less mortality when compared to hypertensive disorders and abortion complications. Zimbabwe should adopt the maternal near miss ratio as an indicator for evaluating its maternal health services. There is need for a nationwide study on the incidence of MNM to rule out regional variability and to include facilities that offer comprehensive emergency obstetric care in the sample. Well-equipped maternity HDUs and ICUs should be established to reduce the MI from obstetric emergencies.

## Abbreviations

HDU: High dependence unit
ICU: Intensive care unit
MD: Maternal deaths
MI: Mortality index
MMR: Maternal mortality ratio
MNM: Maternal near miss
SMO: Severe maternal outcomes
WHO: World Health Organization
